# A compatible interaction of *Alternaria brassicicola *with *Arabidopsis thaliana *ecotype DiG: evidence for a specific transcriptional signature

**DOI:** 10.1186/1471-2229-9-31

**Published:** 2009-03-18

**Authors:** Arup K Mukherjee, Sophie Lev, Shimon Gepstein, Benjamin A Horwitz

**Affiliations:** 1 Division of Plant Biotechnology, Regional Plant Resource Centre, IRC Village, Bhubaneswar 751015, Orissa, India; 2 Department of Biology, Israel Institute of Technology, Technion, Haifa 32000, Israel

## Abstract

**Background:**

The interaction of *Arabidopsis *with *Alternaria brassicicola *provides a model for disease caused by necrotrophs, but a drawback has been the lack of a compatible pathosystem. Infection of most ecotypes, including the widely-studied line Col-0, with this pathogen generally leads to a lesion that does not expand beyond the inoculated area. This study examines an ecotype, Dijon G (DiG), which is considered sensitive to *A. brassicicola*.

**Results:**

We show that the interaction has the characteristics of a compatible one, with expanding rather than limited lesions. To ask whether DiG is merely more sensitive to the pathogen or, rather, interacts in distinct manner, we identified genes whose regulation differs between Col-0 and DiG challenged with *A. brassicicola*. Suppression subtractive hybridization was used to identify differentially expressed genes, and their expression was verified using semi-quantitative PCR. We also tested a set of known defense-related genes for differential regulation in the two plant-pathogen interactions. Several known pathogenesis-related (*PR*) genes are up-regulated in both interactions. *PR1*, and a monooxygenase gene identified in this study, *MO1*, are preferentially up-regulated in the compatible interaction. In contrast, *GLIP1*, which encodes a secreted lipase, and *DIOX1*, a pathogen-response related dioxygenase, are preferentially up-regulated in the incompatible interaction.

**Conclusion:**

The results show that DiG is not only more susceptible, but demonstrate that its interaction with *A. brassicicola *has a specific transcriptional signature.

## Background

*Alternaria brassicicola*, the agent of black spot disease of crucifers, is able to infect *Arabidopsis*. Different ecotypes and genetic backgrounds show variation in susceptibility to this necrotrophic pathogen. Defenses against necrotrophs and biotrophs employ different mechanisms [1]. Programmed cell death and production of reactive oxygen species (ROS) are hallmarks of the hypersensitive response (HR) that is a means of plant defense against biotrophs [2,3]. Perception of the pathogen leads to rapid changes in expression of genes including receptor-like protein kinases, followed by cell death and HR-related defense [4-6]. Necrotrophs, in contrast, assimilate nutrients from dead host tissue, and actually benefit from ROS production and programmed cell death [7,8]. It was found, for example, that oxalic acid is apparently a virulence factor for *Sclerotinia sclerotiorum *because it signals for increased ROS production and programmed cell death in the plant [9]. Studies with *Arabidopsis *mutants in different hormone-dependent defense pathways showed that defense against necrotrophs primarily employs jasmonic acid and ethylene-dependent pathways [10,11]. Integration with SA-dependent pathways is also important, and there is cross-talk between the SA and JA pathways [12-14]. An estimated 0.48% of the *Arabidopsis *transcriptome was induced two-fold or more in response to infection with the wide host-range necrotroph *Botrytis cinerea*, and the expression of these genes depends on ethylene, jasmonate and SA pathways [15]. Defense against necrotrophs thus does not necessarily follow the gene-for-gene pattern in which successful recognition implies triggering of the HR. Inoculation of *Arabidopsis *leaves with *A. brassicicola *generally leads to an incompatible interaction in which the lesion does not spread significantly beyond where the fungus was inoculated. In contrast, *Brassica oleracea *is a compatible host, and spreading necrotic lesions are formed. Extensive gene expression data are available for incompatible interactions between *Arabidopsis *and *A. brassicicola *[16-18]. Incompatible and compatible interactions with the bacterial pathogen *Pseudomonas syringae *were compared in a genome-wide study, and the conclusion was that the distinction is mainly a quantitative and kinetic one [19].

The molecular basis for the extent to which the plant can limit infection by necrotrophic fungi is of obviously of great interest, but the use of *Arabidopsis *genetics to investigate this question has been limited by the need to study compatible and incompatible pathosystems for the same pathogen species. Efforts to overcome this gap have begun for *Colletotrichum*-*Arabidopsis *and *Leptosphaeria*-*Arabidopsis *pathosystems [20,21]. Interactions with species of *Botrytis *have been studied at the cellular level, leading to a model in which resistance depends on the balance between cell death and survival [22]. *A. brassicicola *is an attractive system because of the considerable amount of work already done with this pathosystem. As for *Leptosphaeria *and *Botrytis*, there is a genome project for *A. brassicicola *[23], which is currently in the manual curation stage (Dothidiomycete group and the Joint Genome Institute, US Department of Energy, unpubl.). *Arabidopsis *mutants defective in biosynthesis of the antimicrobial compound camalexin are more susceptible to *A. brassicicola *[24-26]. The Dijon-G (DiG) ecotype is one of the most susceptible, and is a low-camalexin ecotype [27]. Additional factors are involved, because disease resistance did not directly correlate with camalexin levels in the 24 ecotypes studied [27]. Indeed, *A. brassicicola *infection of wild type and camalexin-deficient *pad3 *mutant plants resulted in a generally similar transcriptional pattern [17]. A secreted lipase encoded by the *Arabidopsis *gene *GLIP1 *is important for resistance to *A. brassicicola *[28]. Analysis of the *A. brassicicola*-*Brassica oleracea *interaction led to the identification of a collection of *A. brassicicola *EST sequences characteristic of this compatible interaction [29]. The DiG-*A. brassicicola *pair, chosen to investigate the role of the *A. brassicicola *non-ribosomal peptide synthase gene *NRPS6 *as a virulence factor [30], has a compatible appearance. This led us to consider the question of whether there is merely a continuous range of susceptibility among ecotypes, or rather a fundamental difference between compatible and incompatible interactions. If one postulates that the DiG and Col-0 interactions with *A. brassicicola *differ merely in the extent of sensitivity of the plant to the pathogen, the transcriptional profiles should be very similar in the two interactions. The aim of this study was to ask whether the transcriptional profile of this particular *A. brassicicola*-*Arabidopsis *interaction differs from that of an incompatible interaction. Differential cDNA screening and a candidate gene approach led to the identification of specific markers for the two types of interaction. The hypothesis that the interactions are identical can thus be excluded.

## Results

**Figure 1:**
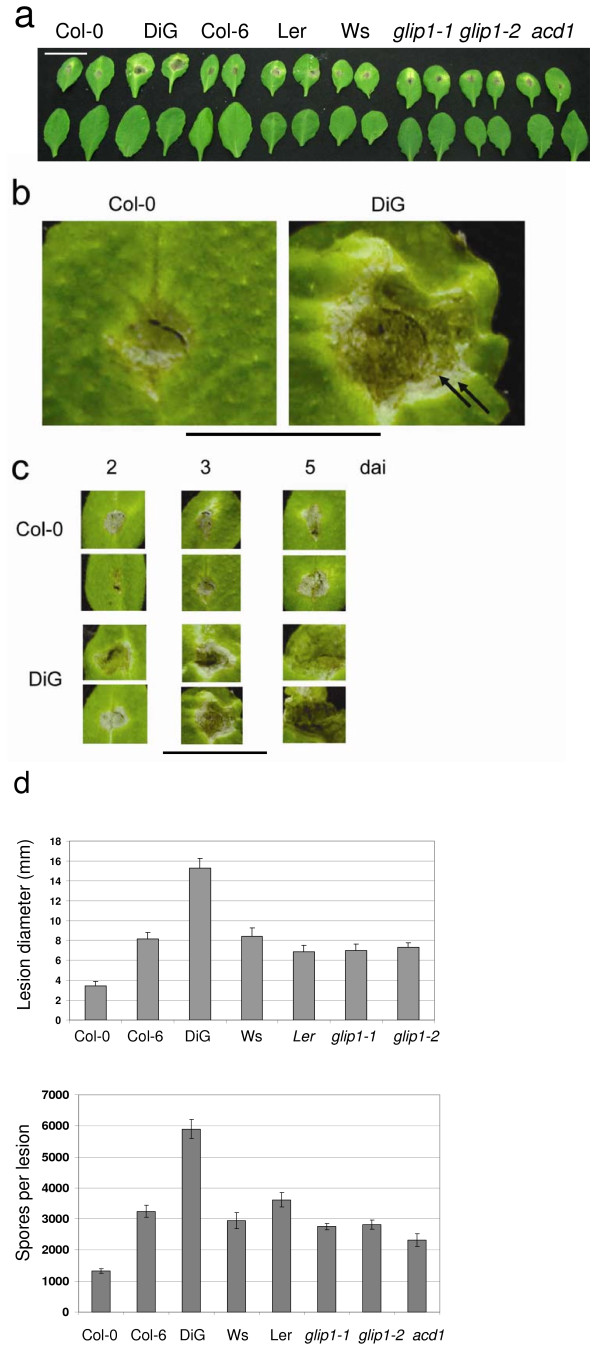
**Characterization of *Arabidopsis-Alternaria brassicicola *pairs**. a) Symptoms in different ecotypes and genotypes, 3 days after inoculation of intact leaves. Top row, inoculated; bottom row, control. *glip1-1 *and *glip1-2 *are two mutants at the *glip1 *locus encoding a secreted lipase [28]; *acd1 *is a lesion mimic mutant [32]. Scale bar indicates 2 cm. b) Magnification of images of leaves from (a) showing the ring-like pattern in the progression of the lesion on a DiG leaf (arrows). The innermost dark, thin, arc (no arrow) is material from the inoculum. Scale bar indicates 1 cm. c) Size of lesions on Col-0 and DiG leaves at different times post-inoculation. Representative infected leaves are shown, photographed at the indicated times after inoculation. Scale bar indicates 2 cm. d) Quantitative analysis of lesion size and spore production. Top panel, lesion diameter was measured 5 days after inoculation. Error bars indicate standard errors of the mean for 7 replicate lesions (for Col-0, 9 and DiG, 10 replicates). Lower panel, lesions were excised 5 days after inoculation, the conidia suspended in water, and counted under the microscope in a hemocytometer chamber. Values are means of two independent experiments, consisting of 12 and 4–5 replicates, respectively; the error bars indicate the standard error of the mean of the combined data from the two experiments.

A total of five wild type ecotypes, Col-0, Col-6, DiG (Dijon G), Ler (Landsberg erecta), Ws (Wassilewskija) and three mutants (*glip1-1*, *glip1-2 *and *acd1*) were screened against *A. brassicicola *(Fig. 1a). These mutants were tested initially, because *glip1 *mutants were shown to be susceptible to *A. brassicicola *[28]. The mutant *acd1 *was chosen because we reasoned that, as *LLS1 *in maize and its ortholog *ACD1 *in *Arabidopsis *are required to limit the spread of cell death [31,32], loss of this gene might increase the spread of a necrotrophic pathogen. Inoculum amount and sampling times after inoculation were calibrated in preliminary experiments so that the different plant-fungal pairs could be compared under non-saturating conditions (data not shown). Lesion diameter and spore production were measured (Fig. 1b) to assess the disease progression. The Col-0 accession showed an incompatible interaction, in which the lesion did not progress beyond the boundaries of the inoculated region. Accession DiG was most susceptible, showing larger lesions than either of the *glip1 *mutants or Col-6, which are relatively susceptible as compared to Col-0 [28]. The lesion-mimic mutant *acd1*, was no more susceptible to the pathogen than was Col-0 (Fig. 1a, d). The lesions on DiG leaves continued to spread (Fig. 1c) and often show concentric rings (Fig. 1b), as seen in the interaction with the compatible host *Brassica oleracea *but not in incompatible interactions with *Arabidopsis*. To test the possibility that the DiG-*A. brassicicola *interaction has a unique transcriptional signature, two approaches were followed: differential library screening and a candidate gene approach.

A suppression-subtractive hybridization (SSH) library was constructed to compare *A. brassicicola*-infected to mock-inoculated leaves of ecotype DiG. In order to limit the set of ESTs selected, to the extent possible, to plant transcripts, cDNA from RNA isolated from a saprophytic culture of *A. brassicicola *was added to the driver population. In the SSH procedure, the driver competes with the differentially expressed transcripts. Furthermore, the library was constructed at 72 h post-infection, when most of the leaf was still green in both plant-fungal pairs (Fig. 1). Sequence was obtained for 116 clones (Table 1). The library was of high diversity: a chitinase clone, for example, was represented 5 times, and a glycosyl hydrolase three times in the sequences, but most transcripts were represented only once (Table 1). Almost all the sequences were identified in the *Arabidopsis *genome database. 14 of the genes in this set were already known to be up-regulated in response to *A. brassicicola *infection [17], and an additional transcript, *PDIOX1*, was also known to be up-regulated in incompatible interactions [33]. Most of the genes, however, had not been identified previously in the interaction of *A. brassicicola *with Col-0 and its mutants. To determine whether these are all false positives or marginally up-regulated genes, or rather, represent a class of genes up-regulated in the compatible interaction, a set of primer pairs was designed based on sequences of nine clones representing transcripts that were not identified [17] in the interactions with Col-0 and its mutants, and which have annotated functions (Table 2). In an additional, candidate gene, approach, a set of known defense-response related genes (Table 2) was tested.

**Table 1:** Randomly isolated SSH clones.

***Annotation***	***TAIR number***	***inc***	***primers***	***x***
60S ribosomal protein L13A	AT3G24830.1	no		
SIR sulfite reductase	AT5G04590.1	yes		3
PSAL photosystem I subunit L	AT4G12800.1	no		
Cobalamin-independent methionine synthase	AT5G17920.2	no	MTH	
glutamine synthase	AT1G66200.1	no	GS	2
alpha dioxygenase 1	AT3G01420.1	no	PDIOX	
NPQ4 non-photochemical quenching	AT1G44575.1	no		
protein phosphorylated amino acid binding	AT5G10450.2	no		2
FF domain-containing protein 14-3-3 stress related	AT3G19670.1	no		2
sorbitol dehydrogenase	AT5G51970.2	no		2
26S proteasome AAA-ATPase subunit	AT5G19990.1	no	PATP	2
no hit				
APX1 ascorbate peroxidase	AT1G07890.7	yes		
acyl-CoA oxidase	AT5G65110.2	no		
chloroplastic drought-induced stress protein	AT1G76080.1	no		
chloroplast-encoded 23S ribosomal	ATCG01180.1	no		2
no hit				
SHM3 serine hydroxymethyltransferase	AT4G32520.1	no		
no hit				
PDF1.2 defensin	AT5G44420.1	yes		
Chitinase	AT2G43590.1	yes		6
no hit				
lipid binding	AT1G04970.2	no		
sugar transporter	AT1G77210.1	no		
no hit				
LHCA4 Photosystem I light harvesting complex gene 4	AT3G47470.1	no		
ribosomal L6	AT1G74050	no		
dehalogenase hydrolase	AT2G32150.1	no		
expressed protein	AT1G07040.1	no		
protein kinase	AT2G23450.1	no		
NADPH cyt P450 reductase	AT4G24520.1	no	P450	
no hit				
GTP binding	AT1G17470.1	no		
GST	AT1G65820.1	no		
RuBisCo	AT5G38410.1	no		2
cobalamin independent methionine synthase	AT3G03780.2	no		
inorganic carbon transport, small stretch	AT4G32340.1	no		
malate dehydrogenase	AT5G09660.2	no	MDH	
lipase class 3	AT5G24210.1	no		
no hits				
glycosyl hydrolase family 17	AT4G16260.1	yes		4
no hits				
peroxidase 42 (PER42)	AT4G21960.1	no	PRX42	
speckle-type POZ protein-related	AT3G48360.1	no		
UDP-glucose 4-epimerase	AT1G12780.1	no		
cysteine proteinase (RD21A)/thio	AT1G47128.1	no	RD21A	
chlorophyll A-B binding	AT3G61470.1	no		
amino acid transporter family	AT3G56200.1	no		
expressed protein	AT5G23040.2	no		
glutamate:glyoxylate aminotransfer	AT1G23310.2	no		
expressed protein	AT5G02020.2	no		
cysteine synthase, putative/O-ac	AT5G28030.1	no		
cytochrome b5 domain-containing	AT3G48890.1	yes		
protein kinase family protein	AT3G51550.1	no		
chlorophyll A-B binding	AT1G61520.2	no		
sugar transport protein (STP4),	AT3G19930.1	no		
expressed protein	AT5G54540.1	no		
glycine hydroxymethyltransferase	AT4G37930.1	no		
similar to gamma-glutamylcysteine	AT4G23100.1	yes		
no hits				
autophagy 7 (APG7)	AT5G45900.1	no		
expressed protein	AT3G15450.3	no		
expressed protein	AT4G19160.3	no		
expressed protein	AT1G02475.1	no		
serine-rich protein-related	AT5G25280.2	no		
GST	AT4G02520.1	yes		
expressed protein	AT1G26110.1	no		
GSH1	AT4G23100.1	yes		
meprin and TRAF homology domain	AT2G32870.1	no		
PSBO2	AT3G50820.1	no		
actin-depolymerizing factor 1	AT3G46010.1	no		
spermidine synthase 2	AT1G70310.1	no		
cytochrome P450 (CYP83B1)	AT4G31500.1	no		2
2-oxoacid-dependent oxidase	AT3G49620.1	no		
dehydrin (RAB18)	AT5G66400.1	no		
expressed protein	AT1G64360.1	no		
auxin-responsive protein	AT3G07390.1	no		
3-oxoacyl-(acyl-carrier protein)	AT1G24360.1	no	3OA	
cysteine proteinase	AT5G60360.2	no		
no hits				
peroxisomal membrane 22 kDa family	AT5G19750.1	no		
CBL-interacting protein kinase 6	AT4G30960.1	no		
coronatine-responsive protein	AT1G19670.1	yes		2
hevein-like protein	AT3G04720.1	yes		
monooxygenase (MO1),	AT4G15760.1	no	MO1	
KELP transcriptional coactivator p15	AT4G10920.1	no		
peroxidase 42 (PER42)	AT4G21960.1	no		
ubiquitin-conjugating enzyme 1	AT1G14400.2	no		
mannitol transporter	AT4G36670	yes		
aspartate aminotransferase 3	AT5G11520.1	no		
basic helix-loop-helix (bHLH) fami	AT5G46760.1	no		
expressed protein	AT4G25030.2	yes		
no apical meristem (NAM) family	AT1G69490.1	no		
expressed protein	AT2G15890.1	no		
TMS membrane family protein	AT1G16180.1	no		
expressed protein	AT5G54730.1	no		
60S ribosomal protein L23A	AT2G39460.1	no		
CBL-interacting protein kinase 6	AT4G30960.1	no		
Calmodulin	AT2G41410.1	yes		
RER1B	AT2G21600.1	no		
glycine-rich RNA-binding protein	AT4G13850.2	no		
chlorophyll A-B binding	AT2G05070.1	no		
cytochrome C	AT1G22840	yes		

The nucleotide sequences were used to search the TAIR database by BLASTN. A brief annotation and link to the TAIR database are given for each sequence. "No hits" indicates that no *Arabidopsis *gene was identified; these transcripts might represent fungal genes. The test primer pairs chosen are listed (names refer to Table 2), as well as the number of times (x) that the same gene was identified in the set of cDNA clones sequenced (if more than once). Clones marked "yes" were previously reported [17] in an incompatible *Arabidopsis-Alternaria *interaction (inc).

**Table 2:** Test primer pairs used for semi quantitative RT-PCR amplification: names, TAIR database numbers, and predicted product sizes in bp are listed.

***name***	***function***	***TAIR ID***	***sense primer***	***antisense primer***	***bp***
PATP	proteasome subunit	AT5G19990	GGCGTCCTGAGACAGCGATGGAG	CAGGCCTGAGAAGAGCTTGATCCAG	938
GS	cytosolic glutamine synthetase	AT1G66200	GAAGGATGTGAACTGGCCTCTTG	GTAAGGGTCCATGTTTGAAGCTG	615
PDIOX	pathogen inducible dioxygenase	AT3G01420	GTATGCGACGCCCTCAAGGATG	CCTTGAGACTCTCTGTAGTATTCACC	936
MTH	homocysteine methyltransferase	AT5G17920	GCTGATCTCAGGTCATCCATCTG	GATTGAGCTTCTTCTGCTGAGCATC	1170
MDH	malate dehydrogenase	AT5G09660	GGAAAACTGCAGAGCTAAAGGTGG	CCAAGCTGATACACTTCCTCTGC	878
PRX42	peroxidase 42	AT4G21960	GACCACAACGAGAGTATCTCCGTC	CAAGCAGAGAACTCAACACACCAGAG	544
RD21A	cysteine proteinase (RD21A)	AT1G47128	GTGAGAGAAGGACTAGCCTACGGTAC	CACAAACCGGGTACTCGTGAG	914
3OA	3-oxoacyl-(acyl-carrier protein)	AT1G24360	GATGAAAACCGCTCTTGACAAATG	CATCAATGGTGAATGCCTGTCC	511
MO1	aromatic-ring hydroxylase	AT4G15760	GTTTGCTTGTCGTGCGGTGAGAG	CTGCACCGAAAGCCCGAGTAATC	555
PR1	PR1 pathogen related	AT2G14610	TCGTCTTTGTAGCTCTTGTAGGTG	TAGATTCTCGTAATCTCAGCTCT	590
GLIP1	lipase, defense response	AT5G40990	CGATTGTGCACCAGCCTCATTGGTT	CAGCGCTTTGAGATTATAGGGTCC	429
PR2	PR2 pathogen-related, cellulase	AT3G57260	CGTTGTGGCTCTTTACAAACAACAAAAC	GAAATTAACTTCATACTTAGACTGTCGAT	870
PR3	PR3 defense response chitinase	AT3G12500	CGGTGGTACTCCTCCTGGACCCACCGGC	CGGCGGCACGGTCGGCGTCTGAAGGCTG	583
PR4	PR4 hevein-like defense protein	AT3G04720	GACAACAATGCGGTCGTCAAGG	AGCATGTTTCTGGAATCAGGCTGCC	552
PR5	PR5 thaumatin-like	AT1G75040	ATGGCAAATATCTCCAGTATTCACA	ATGTCGGGGCAAGCCGCGTTGAGG	484
PDF1.2	defensin	AT5G44420	GCTAAGTTTGCTTCCATCATCACCCTT	AACATGGGACGTAACAGATACACTTGT	237
EIN2	ethylene signal transduction	AT5G03280	TGCAGCTCGCATAAGCGTTGTGACTGGTA	CGCTCTCTCCATTTAACCGAGTTAACAC	379
EIN3	ethylene signal transduction	AT3G20770	GATGTTGATGAATTGGAGAGGAGGATG	ACGTCTCTGAGGAGGATCACAGTGT	470
ERF1	ethylene response factor	AT3G23240	CGGCTTCTCACCGGAATATTCTATCG	TCTCCGAAAGCGACTCTTGAACTCTCT	415
ACT2	actin 2	AT3G18780	TCACCACAACAGCAGAGCGGG	GGACCTGCCTCATCATACTCGG	257
UBC	ubiquitin conjugating	AT5G25760	GGCATCAAGAGCGCGACTGTT	CTTTCTTAGGCATAGCGGCGAG	217
CBP20	Cap-binding protein 20	AT5G44200	TTGTGGCTTTTGTTTCGTCCTG	CGTGGGTTCTTCTCCGGTCTC	409

**Figure 2:**
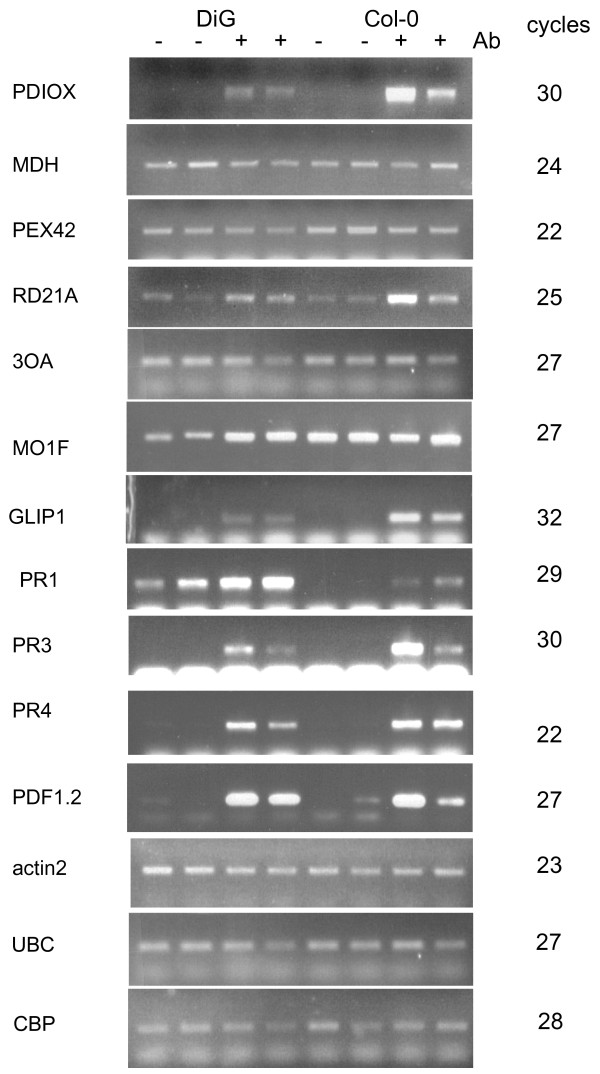
**Semi-quantitative RT PCR analysis of transcript levels of selected genes**. - and + indicate samples from control and inoculated intact plants, respectively. RNA was isolated by harvesting the entire leaf at 72 h after inoculation. Duplicate lanes indicate two independent experiments on different sets of plants; the number of amplification cycles is indicated at the right.

**Figure 3:**
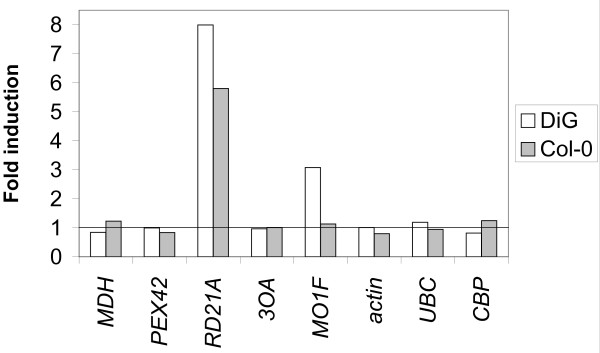
**Expression of the monooxygenase gene *MO1 *is preferentially up-regulated in the compatible interaction**. Relative transcript levels were calculated from the intensity of the RT-PCR signals shown in Figure 2, as follows. Infected Col-0 was chosen as the reference treatment. The band intensities of the three reference genes (*ACT2*, *UBC *and *CBP20*) then showed similar expression patterns as a function of experiment and replicate, over the entire data set, with no clear trend as a function of treatment. This indicates that the transcript levels of these genes varied with the amount of RNA and efficiency of the reactions, rather than with the treatment. All data for the reference genes were therefore combined, and the entire data set normalized to the combined reference values to obtain the signal plotted as "fold induction" (y-axis).

Semi-quantitative RT-PCR analysis of the abundance of the corresponding transcripts is shown in Fig. 2. An actin gene (*ACT2*), a ubiquitin-conjugating enzyme gene (*UBC*) and cap-binding protein 20 (*CBP20*) were used as "housekeeping" genes (Table 2). The false-positive library clones also serve as additional controls for overall efficiency of the RT PCR procedure (Fig. 2). The fold-induction by infection relative to Col-0 is shown in Fig. 3. The transcripts detected by three of the test primer pairs: PDIOX, RD21A and MO1F (Table 2) showed clearly differential expression in the compatible interaction. The transcripts corresponding to GS, MDH and PEX42 did not, although even a slight differential expression might have led to inclusion in the library. The transcript corresponding to primer pair PDIOX is more highly expressed in the incompatible interaction. The relatively low proportion of differential SSH clones in the test set (3 out of 9) may reflect the choice of test primer pairs, which included only genes that were not previously annotated as pathogen-response dependent (Table 1). Among the candidate genes tested, *PR1*, *PR3*, *PR4 *and *PDF1.2 *were strongly up-regulated in both Col-0 and DiG interactions. The transcript levels of these genes were very low in uninfected plants, with the exception of *PR1 *(Fig. 2), so that induction ratios could not be estimated. Transcripts of the other candidates (Table 1), including three ethylene-response related genes, were not detectable under these conditions and it can be inferred that they are not strongly induced. The infection-related lipase gene *GLIP1 *is expressed at a higher level (about 4-fold) in the incompatible than in the compatible interaction, while *PR1 *is expressed about 4-fold higher in the compatible than in the incompatible interaction.

## Discussion

The *A. brassicicola*-DiG pathosystem has the features of a compatible interaction, producing expanding necrotic lesions. This suggests that there may be a fundamental difference between this interaction and an incompatible one, rather than merely a graded increase in sensitivity relative to Col-0. If this is so, the defense responses of the plant should differ between the compatible and incompatible interactions. As extensive transcriptional profiling has already been reported for incompatible *A. brassicicola*-*Arabidopsis *interaction (incompatible), an initial study of the *A. brassicicola*-DiG pathosystem was performed. The set of transcripts detected overlaps partially with those induced in resistant (Col-0) or relatively sensitive (*pad3 *in Col-0 background) interactions, but most of the SSH clones represent transcripts that had not been identified before as defense-related. Of a test set of 9 genes from the SSH library tested by RT-PCR, three were differentially expressed at 72 hai in the DiG-*A. brassicicola *interaction: the primer pair PDIOX (Fig. 2) corresponds to a gene which encodes an alpha-dioxygenase involved in protection against oxidative stress and cell death, and induced in response to salicylic acid and oxidative stress.

This gene, *DIOX1*, is preferentially induced in the incompatible interaction with *A. brassicicola*, in agreement with previous data for *Arabidopsis*-bacteria interactions [33]. Induction of the monooxygenase/aromatic-ring hydroxylase gene *MO1 *is specific to the compatible interaction. In Col-0 *MO1 *is not induced, and is present already in non-infected plants. This suggests that Col-0 may be "primed" in some way to initiate the defense response that is characteristic of infection with *A. brassicicola*. *MO1 *shares homology with monooxygenases that degrade SA. Overexpression of the SA-degrading enzyme NahG has been used to test the involvement of SA in defense responses, but the immediate product, catechol, may contribute to the phenotypes of NahG expressors [34]. The reaction catalyzed by Mo1 is not known, but one possibility is that this enzyme produces SA-derived aromatic compounds that could have signaling roles. Another possibility is that Mo1 might be involved in the suppression of the SA pathway in Col-0. It is worthy of note that *PR1 *is more highly expressed in DiG, the opposite of what would be expected if Mo1 acts like NahG. *GLIP1 *shows the reverse pattern, and is induced less in the compatible interaction. This extracellular lipase-related protein contributes to resistance to *A. brassicicola *[28]. The decreased ability of DiG to upregulate *GLIP1 *in response to *A. brassicicola *may therefore directly contribute to its sensitivity to the pathogen. DiG is a low producer of camalexin but this is probably not the only reason for its sensitivity, since other susceptible ecotypes produced up to several fold more camalexin than Col-0 [27]. Furthermore, the expression profiles of camalexin-lacking *pad3 *and wild type (Col-0) were similar and the data sets were indeed combined [17]. This contrasts with what was found here for DiG, suggesting that additional heritable traits are involved. Segregation of incompatible interaction – related traits in crosses between DiG and Col-0 may identify loci other than those encoding camalexin biosynthesis genes. One candidate is *GLIP1*, and it would be of interest to construct a mutant lacking both *GLIP1 *and camalexin. Another candidate is the alpha dioxygenase *DIOX1*; the oxylipin signals produced by the alpha-DOX1 fatty acid dioxygenase encoded by this gene promote protection from ROS and cell death [33]. *PR1*, in contrast, is expressed at higher levels in DiG, despite the fact that this is a gene strongly induced by the SA pathway. This suggests that in DiG, the SA pathway might be induced upon infection with a necrotroph. Induction of the SA and JA pathways is coordinated, with induction of one pathway at the expense of the other [13,14]. Infection by a biotroph suppresses defense against the necrotroph *A. brassicicola*. Furthermore, the application of SA resulted in suppression of defense against the necrotroph, and high expression of the SA-dependent defense gene *PR1 *[14]. The JA pathway is most important for defense against *A. brassicicola *[10]. Thus, induction of the SA pathway might be an important factor responsible for the development of a compatible interaction with DiG. It is possible that the extent of the trade-off between SA and JA-dependent pathways [14] has been modulated by selection in different plant ecotypes. It is striking that application of SA [14] closely mimicked the appearance of the compatible-type lesions that we observed in ecotype DiG (Fig. 1A–C). Likewise, we found strong induction of *PR1 *expression (Fig. 2). Expression of the JA-dependent defensin gene *PDF1.2*, however, was not strongly suppressed in DiG (Fig. 2), while application of SA strongly suppressed PDF1.2 expression over the entire 3-days post-inoculation period studied in [14]. Thus, suppression of JA-mediated defense may be only part of the explanation of the susceptible phenotype of DiG. An alternative explanation is that the reciprocal regulation of the SA and JA pathways might fail as a result of successful infection by the pathogen, making this an effect, rather than cause, of the difference between the ecotypes. Genes induced in infected as compared to control leaves (in both ecotypes) may have roles in defense responses, or be induced as a result of tissue damage and cell death. The set of ESTs identified by SSH includes known defense-regulated genes. These were not further tested for differential expression here, since they have been previously studied. These ESTs include: chitinase (At2g43590), glycosyl hydrolase family 17 (AT4G16260.1), sulfite reductase/ferredoxin (At5g04590.1), PDF2.1a (At5g44420.1), APX1 – ascorbate peroxidase (At1g07890.1), cytochrome b5 domain-containing (AT3G48890.1), GST (AT4G02520.1), GSH1 (AT4G23100.1), coronatine responsive protein (chlorophyllase, methyl jasmonate induced, AT1G19670.1), hevein-like protein (HEL) (AT3G04720.1), mannitoltransporter (AT4G36670), calmodulin (AT2G41410.1), and cytochrome c (AT1G22840) (Table 1). Our identification of 15 known defense-regulated genes (about 15%, taking into account redundancy in the set of sequenced clones) in the library (Table 1) shows that the SSH comparison was robust, despite only one third of the test set showing clearly differential expression between Col-0 and DiG (Fig. 2). We note that known *A. brassicicola *induced genes were excluded from the test set (Table 2). Although some sequences were redundant in the sample of more than 100 clones, the number of SSH-derived ESTs apparently was not saturated with respect to the number of clones sequenced. PDF1.2, for example, was strongly induced (Fig. 2) but not found among the sequenced clones.

## Conclusion

The DiG-*Alternaria brassicicola *pathosystem shows all the characteristics of a compatible interaction between the necrotroph *A. brassicicola *and *Arabidopsis*. This initial study demonstrates that the transcriptional profile of the compatible interaction is not identical to that of the well-studied incompatible interaction with Col-0. Induction of the monooxygenase gene *MO1*, and high expression and induction of *PR1*, are characteristic of the compatible interaction. *GLIP1 *and *DIOX1*, in contrast, are expressed more strongly in the incompatible interaction. These particular genes are not necessarily those whose expression levels define whether the plant is able to limit lesion spread or not, but are candidates for further study. The similarity between the compatible interaction and the result of exogenous application of SA provides a clue to the mechanism. Full-scale genome-wide studies are being done for the interaction of the hemibiotroph *Magnaporthe oryzae *with rice cultivars providing compatible or incompatible interactions [35,36]. This approach can now be fully developed for the *Arabidopsis-Alternaria *pathosystem defined in this study.

## Methods

### Plant material, growth conditions and RNA extraction

Seeds of the ecotypes Col-0, Col-6, WS and DiG were obtained from ABRC . Mutants *glip1-1*, *glip1-2 *and *acd1 *were from the same collection, and homozygous lines were selected by screening progeny of selfed plants by PCR on genomic DNA samples using appropriate diagnostic primer pairs. Seeds were sown in "cookies" (approximately 4 cm diameter soil packaged in netting, purchased locally) held at 4°C for two days to promote germination, and plants were grown in a temperature-controlled room at 23°C under continuous or 16 h/8 h fluorescent lighting (cool-white tubes). Rosette leaves were inoculated with a conidial suspension of *Alternaria brassicicola *(MUCL20297, [10]). Inoculation was as described in [17], except that in preliminary experiments we found that a higher inoculum was needed to obtain reproducible disease development on both ecotypes, and so used 5 μl of a 6 × 10^6 ^spore/ml suspension (3 × 10^4 ^conidia per drop) throughout this study. To prepare spores for inoculation, fungal cultures were grown in a growth chamber on potato dextrose agar (Difco) plates under continuous white light at 25°C for 7 days, and spores suspended in water and counted in a haemocytometer. Plants were inoculated in a biosafety laminar flow chamber, placed in large sealed containers and incubated for up to 5 days at 23–24°C in a growth chamber. Conidia production was assayed after 5 days following inoculation by suspending the conidia from lesions excised from 10 infected leaves as described [17]. Leaf material was harvested, frozen, and kept at -80°C until further use. RNA was extracted from control (mock inoculated) and inoculated leaves of Col-0 and DiG using Tri-Reagent (MBC or Fluka) according to the manufacturer's protocol, except that the starting material was leaf tissue ground in liquid nitrogen. Each sample for RNA extraction consisted of about 20 leaves from at least 10 plants. (All the experiments were repeated at least three times with three replications) RNA concentrations were determined using a Nanodrop spectrophotometer and quality was checked by electrophoresis on denaturing agarose gels.

### Suppression-subtractive hybridization (SSH)

4 week old DiG plants were inoculated with *A. brassicicola *with at least three replications and three biological repetitions. The infected leaves were collected after 6 h (Ast-1), 12 h (Ast-2), 24 h (Ast-3), 48 h (Ast-4) and 72 h (Ast-5) of inoculation. Total RNA was isolated from each stage of disease development along with control (Ast-0). The leaf samples were pooled for each stage from each replication and repetition of the experiment. For the SSH analysis the mRNA was enriched from the total RNA of Ast-0, Ast-3, Ast-4 and Ast-5 using the Qiagen Oligotex mRNA isolation Kit. SSH was performed using the Clontech PCRSelect cDNA subtraction kit following the manufacturer's protocol and the driver population consisted of mRNA from Ast-0 and *A. brassicicola *in the ratio of 3:1. *A. brassicicola *RNA was from a culture grown for 60 hours in shake culture on PDB. Amplification using primer pairs specific for the defense response gene *PR1 *and an actin gene confirmed that the SSH procedure functioned as expected (data not shown). For this analysis, the following primers were used, spanning regions without *Rsa*I sites: *PR1 *primer, sense direction, see Table 2; *PR1 *antisense, GATCACATCATTACTTCATTAGTATG. *ACT2 *primers: sense GCTGGATTCTGGTGATGGTG, antisense GATTCCAGCAGCTTCCATTC.

An enrichment of 64 fold for *PR1 *relative to *ACT2 *was estimated from these amplification data; it should be noted that this is likely to be the combined effect of suppression of *ACT2 *cDNA abundance and/or enrichment of *PR1*, as expected from the design of the SSH subtraction method (see PCRselect manual, Promega, and literature cited therein). The fragments amplified in the second PCR reaction were cloned into pTZ57R/T (Fermentas), transformed into *E. coli *DH5α (HIT, Real Biotech), 130 positive clones were picked and the inserts amplified from the bacterial colonies using M-13 forward and reverse primers. Sequence was obtained for 116 clones (Macrogen, Seoul, Korea).

### Semiquantitative RT PCR analysis

cDNA was synthesized and assayed as follows. 2 μg of total RNA from rosette leaves of DiG or Col-0 plants inoculated as described above were treated with 2 units of RQ1 RNAse-free DNAse (Promega) in a volume of 10 μl. After addition of stop solution and incubation for 10 min at 65°C, the sample was denatured in the presence of 0.5 μg of oligo dT primer, cooled, 200 units of MMLV reverse transcriptase (Promega), 24 units of PRI RNAse inhibitor (PRI, TaKaRa) and dNTPs to a final concentration of 0.5 mM each were added, and the reaction volume adjusted to a total of 25 μl in 1× MMLV reaction buffer. cDNA synthesis was for 1 h at 42°C. All reactions were carried out in a thermal cycler (Biometra). A set of three "housekeeping" gene primer pairs (Sigma) was used to calibrate template amount (Table 2). The cDNA samples were diluted such that similar signal intensity was obtained upon amplification with the *Actin 2 *(*ACT2*) primer pair (Table 2), and the number of cycles was calibrated for each primer pair in order for the amplification level to remain below saturation.

## Abbreviations

dai: days after inoculation; hai: hours after inoculation; ROS: reactive oxygen species; HR: hypersensitive response; SA: salicylic acid; JA: jasmonic acid

## Authors' contributions

AKM conceived of the study together with the other authors, and carried out the major part of the experiments. SL brought the compatible interaction phenotype of DiG to the attention of AKM and BAH, and participated in library construction and data analysis. SG participated in coordination and analysis of the results. BAH drafted the manuscript and carried out some of the gene expression experiments. All authors participated in writing the final manuscript. All authors read and approved the final manuscript.

## Author information

AKM is a plant molecular geneticist interested in plant pathology and stress physiology. SG leads a group studying gene expression and control of leaf senescence and stress responses in *Arabidopsis *and other plants. BAH lab focuses on signal transduction genes of filamentous fungi including Dothidiomycete pathogens of plants. SL, former member of BAH lab and currently a postdoc at UC Berkeley, studies plant-pathogen interactions by molecular genetic approaches.
